# *N*-(2-Chloro­benzo­yl)-4-methyl­benzene­sulfonamide

**DOI:** 10.1107/S1600536812015681

**Published:** 2012-04-18

**Authors:** P. A. Suchetan, Sabine Foro, B. Thimme Gowda

**Affiliations:** a Department of Chemistry, Mangalore University, Mangalagangotri 574 199, Mangalore, India; b Institute of Materials Science, Darmstadt University of Technology, Petersenstrasse 23, D-64287 Darmstadt, Germany

## Abstract

In the title compound, C_14_H_12_ClNO_3_S, the C=O bond is *syn* to the Cl substituent in the adjacent benzene ring. The C—S—N—C torsion angle is −80.6 (6)°. The chloro­benzoyl ring is disordered and was refined using a split model [occupancy ratio 0.537 (3):0.463 (3)]. In the crystal, mol­ecules are linked by pairs of N—H⋯O(S) hydrogen bonds, forming inversion dimers.

## Related literature

For our studies on the effects of substituents on the structures and other aspects of *N*-(ar­yl)-amides, see: Gowda *et al.* (2000 ▶, 2007 ▶), of *N*-(substitutedbenzo­yl)-aryl­sulfonamides, see: Gowda *et al.* (2010 ▶), of *N*-chloro­aryl­amides, see: Jyothi & Gowda (2004 ▶) and of *N*-bromo­aryl­sulfonamides, see: Usha & Gowda (2006 ▶).
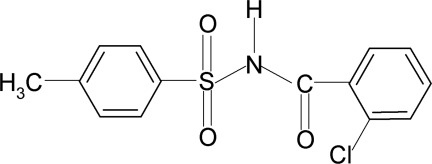


## Experimental

### 

#### Crystal data

C_14_H_12_ClNO_3_S
*M* *_r_* = 309.76
Monoclinic, 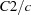

*a* = 25.079 (4) Å
*b* = 8.1963 (7) Å
*c* = 18.397 (3) Å
β = 131.77 (1)°
*V* = 2820.4 (7) Å^3^
*Z* = 8
Mo *K*α radiation
μ = 0.42 mm^−1^
*T* = 293 K
0.48 × 0.20 × 0.16 mm

#### Data collection

Oxford Diffraction Xcalibur diffractometer with a Sapphire CCD detector
Absorption correction: multi-scan (*CrysAlis RED*; Oxford Diffraction, 2009 ▶) *T*_min_ = 0.822, *T*_max_ = 0.935
5253 measured reflections
2432 independent reflections
1623 reflections with *I* > 2σ(*I*)
*R*_int_ = 0.033

#### Refinement

*R*[*F* ^2^ > 2σ(*F* ^2^)] = 0.071
*wR*(*F* ^2^) = 0.115
*S* = 1.16
2432 reflections
216 parameters
15 restraints
H-atom parameters constrained
Δρ_max_ = 0.30 e Å^−3^
Δρ_min_ = −0.31 e Å^−3^

### 

Data collection: *CrysAlis CCD* (Oxford Diffraction, 2009 ▶); cell refinement: *CrysAlis RED* (Oxford Diffraction, 2009 ▶); data reduction: *CrysAlis RED*; program(s) used to solve structure: *SHELXS97* (Sheldrick, 2008 ▶); program(s) used to refine structure: *SHELXL97* (Sheldrick, 2008 ▶); molecular graphics: *PLATON* (Spek, 2009 ▶); software used to prepare material for publication: *SHELXL97*.

## Supplementary Material

Crystal structure: contains datablock(s) I, global. DOI: 10.1107/S1600536812015681/nc2273sup1.cif

Structure factors: contains datablock(s) I. DOI: 10.1107/S1600536812015681/nc2273Isup2.hkl

Supplementary material file. DOI: 10.1107/S1600536812015681/nc2273Isup3.cml

Additional supplementary materials:  crystallographic information; 3D view; checkCIF report

## Figures and Tables

**Table 1:** Hydrogen-bond geometry (Å, °)

*D*—H⋯*A*	*D*—H	H⋯*A*	*D*⋯*A*	*D*—H⋯*A*
N1—H1*N*⋯O2^i^	0.86	2.02	2.867 (4)	169

Symmetry code: (i) 
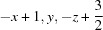
.

**Fig. 1.**
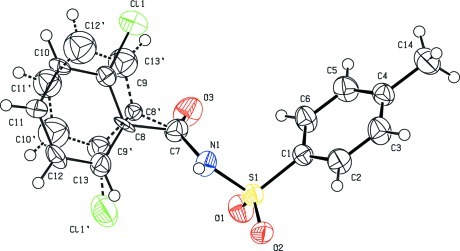
Molecular structure of the title compound, showing the atom- labelling scheme. Displacement ellipsoids are drawn at the 50% probability level and disordering is shown as full and dashed lines.

**Fig. 2.**
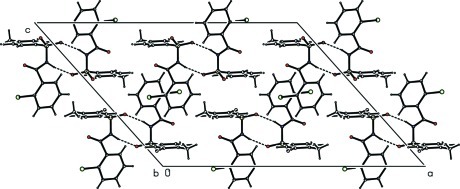
Molecular packing in the title compound. Hydrogen bonds are shown as dashed lines and the disordering is not shown for clarity.

## supplementary crystallographic information

### Comment

As part of our studies on the substituent effects on the structures and other aspects of N-(aryl)-amides (Gowda et al., 2000, 2007), N-(substitutedbenzoyl)-arylsulfonamides (Gowda et al., 2010), N-chloroarylsulfonamides (Jyothi & Gowda, 2004) and N-bromoarylsulfonamides (Usha & Gowda, 2006), in the present work, the crystal structure of N-(2-chlorobenzoyl)-4-methylbenzenesulfonamide (I) has been determined. The conformation of the N—H bond in the C—SO_2_—NH—C(O) segment is anti to the C=O bond (Fig. 1), similar to that observed in i>N-(2-chlorobenzoyl)-4-chlorobenzenesulfonamide (II)(Gowda et al., 2010). Further, the conformation of the C=O bond in the C—SO_2_—NH—C(O) segment of (I) is syn to the ortho-Cl in the benzoyl ring, similar to that observed between in (II).

The molecules are twisted at the S atom with the torsional angle of -80.6 (6)°, compared to that of 65.7 (2)° in (II).

The dihedral angle between the sulfonyl benzene ring and the —SO_2_—NH—C—O segment is 65.0 (5)°, compared to the value of 88.5 (1)° in (II).

Furthermore, the dihedral angle between the sulfonyl and the benzoyl benzene rings is 66.1 (2)°, compared to the value of 58.2 (1)° in (II).

The packing of molecules linked by of N—H···O(S) hydrogen bonds(Table 1) is shown in Fig. 2.

### Experimental

The title compound was prepared by refluxing a mixture of 2-chlorobenzoic acid, 4-methylbenzenesulfonamide and phosphorous oxy chloride for 3 h on a water bath. The resultant mixture was cooled and poured into ice cold water. The solid obtained was filtered, washed thoroughly with water and then dissolved in sodium bicarbonate solution. The compound was later reprecipitated by acidifying the filtered solution with dilute HCl. It was filtered, dried and recrystallized.

Prism like colourless single crystals of the title compound used in X-ray diffraction studies were obtained by slow evaporation of the solvent in its toluene solution at room temperature.

### Refinement

The H atoms were positioned with idealized geometry using a riding model with C—H distances of 0.93 Å (C-aromatic) and 0.96 Å (*C*-methyl) and N—H = 0.86 (2) %A.

*U*_iso_(H) values were set at 1.2 *U*_eq_(C-aromatic, N) and 1.5 *U*_eq_(*C*-methyl).

The chlorobenzyol ring with atoms C8, C9, C10, C11, C12, C13 and CL1 is disordered and was refined using a split model. The corresponding site-occupation factors were refined so that their sum was unity [0.536 (4)–0.464 (4)]. The corresponding bond distances in the disordered groups were restrained to be equal. The C atoms of lower occupancy were refined isotropic.

### Crystal data

**Table:**

C_14_H_12_ClNO_3_S	*F*(000) = 1280
*M**_r_* = 309.76	*D*_x_ = 1.459 Mg m^−^^3^
Monoclinic, *C*2/*c*	Mo *K*α radiation, λ = 0.71073 Å
Hall symbol: -C 2yc	Cell parameters from 2528 reflections
*a* = 25.079 (4) Å	θ = 2.5–27.9°
*b* = 8.1963 (7) Å	µ = 0.42 mm^−^^1^
*c* = 18.397 (3) Å	*T* = 293 K
β = 131.77 (1)°	Prism, colourless
*V* = 2820.4 (7) Å^3^	0.48 × 0.20 × 0.16 mm
*Z* = 8	

### Data collection

**Table:**

Oxford Diffraction Xcalibur diffractometer with a Sapphire CCD detector	2432 independent reflections
Radiation source: fine-focus sealed tube	1623 reflections with *I* > 2σ(*I*)
Graphite monochromator	*R*_int_ = 0.033
Rotation method data acquisition using ω and phi scans	θ_max_ = 25.0°, θ_min_ = 2.7°
Absorption correction: multi-scan (*CrysAlis RED*; Oxford Diffraction, 2009)	*h* = −23→29
*T*_min_ = 0.822, *T*_max_ = 0.935	*k* = −9→7
5253 measured reflections	*l* = −21→19

### Refinement

**Table:**

Refinement on *F*^2^	Primary atom site location: structure-invariant direct methods
Least-squares matrix: full	Secondary atom site location: difference Fourier map
*R*[*F*^2^ > 2σ(*F*^2^)] = 0.071	Hydrogen site location: inferred from neighbouring sites
*wR*(*F*^2^) = 0.115	H-atom parameters constrained
*S* = 1.16	*w* = 1/[σ^2^(*F*_o_^2^) + (0.0079*P*)^2^ + 10.754*P*] where *P* = (*F*_o_^2^ + 2*F*_c_^2^)/3
2432 reflections	(Δ/σ)_max_ = 0.002
216 parameters	Δρ_max_ = 0.30 e Å^−^^3^
15 restraints	Δρ_min_ = −0.31 e Å^−^^3^

### Special details

**Table:**

Experimental. Absorption correction: CrysAlis RED (Oxford Diffraction, 2009) Empirical absorption correction using spherical harmonics, implemented in SCALE3 ABSPACK scaling algorithm.
Geometry. All e.s.d.'s (except the e.s.d. in the dihedral angle between two l.s. planes) are estimated using the full covariance matrix. The cell e.s.d.'s are taken into account individually in the estimation of e.s.d.'s in distances, angles and torsion angles; correlations between e.s.d.'s in cell parameters are only used when they are defined by crystal symmetry. An approximate (isotropic) treatment of cell e.s.d.'s is used for estimating e.s.d.'s involving l.s. planes.
Refinement. Refinement of *F*^2^ against ALL reflections. The weighted *R*-factor *wR* and goodness of fit *S* are based on *F*^2^, conventional *R*-factors *R* are based on *F*, with *F* set to zero for negative *F*^2^. The threshold expression of *F*^2^ > σ(*F*^2^) is used only for calculating *R*-factors(gt) *etc*. and is not relevant to the choice of reflections for refinement. *R*-factors based on *F*^2^ are statistically about twice as large as those based on *F*, and *R*- factors based on ALL data will be even larger.

### Fractional atomic coordinates and isotropic or equivalent isotropic displacement parameters (Å^2^)

**Table:**

	*x*	*y*	*z*	*U*_iso_*/*U*_eq_	Occ. (<1)
S1	0.46756 (6)	0.21314 (14)	0.84045 (8)	0.0370 (3)	
O1	0.47071 (16)	0.0832 (4)	0.8932 (2)	0.0501 (8)	
O2	0.53214 (14)	0.2614 (4)	0.8617 (2)	0.0451 (8)	
O3	0.31747 (17)	0.0536 (4)	0.7036 (2)	0.0606 (10)	
N1	0.41113 (17)	0.1593 (4)	0.7243 (2)	0.0392 (9)	
H1N	0.4221	0.1892	0.6909	0.047*	
C1	0.4310 (2)	0.3870 (5)	0.8486 (3)	0.0331 (10)	
C2	0.4617 (2)	0.5370 (5)	0.8639 (3)	0.0453 (12)	
H2	0.5004	0.5468	0.8677	0.054*	
C3	0.4343 (2)	0.6723 (6)	0.8737 (3)	0.0494 (12)	
H3	0.4547	0.7741	0.8840	0.059*	
C4	0.3767 (2)	0.6586 (6)	0.8682 (3)	0.0408 (11)	
C5	0.3469 (2)	0.5060 (6)	0.8526 (3)	0.0475 (12)	
H5	0.3079	0.4957	0.8481	0.057*	
C6	0.3739 (2)	0.3701 (6)	0.8436 (3)	0.0452 (12)	
H6	0.3540	0.2680	0.8343	0.054*	
C7	0.3475 (2)	0.0709 (5)	0.6741 (3)	0.0406 (11)	
C8	0.3257 (5)	−0.0153 (13)	0.5801 (7)	0.030 (3)	0.537 (3)
C9	0.2525 (4)	−0.0070 (12)	0.4939 (6)	0.031 (2)	0.537 (3)
C10	0.2260 (5)	−0.0831 (12)	0.4066 (6)	0.031 (2)	0.537 (3)
H10	0.1780	−0.0830	0.3496	0.037*	0.537 (3)
C11	0.2799 (5)	−0.1586 (11)	0.4153 (6)	0.037 (2)	0.537 (3)
H11	0.2667	−0.2112	0.3605	0.044*	0.537 (3)
C12	0.3534 (6)	−0.1611 (11)	0.5014 (7)	0.043 (2)	0.537 (3)
H12	0.3859	−0.2151	0.5011	0.052*	0.537 (3)
C13	0.3767 (6)	−0.0868 (14)	0.5831 (8)	0.040 (3)	0.537 (3)
H13	0.4249	−0.0839	0.6389	0.048*	0.537 (3)
Cl1	0.18345 (12)	0.0821 (3)	0.48116 (17)	0.0546 (8)	0.537 (3)
C8'	0.3105 (7)	0.0155 (17)	0.5785 (9)	0.024 (4)*	0.463 (3)
C9'	0.3503 (7)	−0.090 (2)	0.5695 (10)	0.041 (5)*	0.463 (3)
C10'	0.3175 (8)	−0.158 (2)	0.4767 (11)	0.075 (6)*	0.463 (3)
H10'	0.3420	−0.2235	0.4663	0.089*	0.463 (3)
C11'	0.2470 (8)	−0.118 (2)	0.4041 (11)	0.061 (6)*	0.463 (3)
H11'	0.2244	−0.1571	0.3421	0.073*	0.463 (3)
C12'	0.2056 (8)	−0.0234 (18)	0.4137 (11)	0.083 (5)*	0.463 (3)
H12'	0.1573	−0.0046	0.3610	0.099*	0.463 (3)
C13'	0.2385 (8)	0.0399 (19)	0.5032 (11)	0.066 (5)*	0.463 (3)
H13'	0.2124	0.0995	0.5134	0.079*	0.463 (3)
Cl1'	0.43803 (18)	−0.1591 (4)	0.6657 (3)	0.0793 (13)	0.463 (3)
C14	0.3471 (3)	0.8079 (6)	0.8782 (3)	0.0576 (14)	
H14A	0.3478	0.8981	0.8457	0.086*	
H14B	0.3757	0.8334	0.9460	0.086*	
H14C	0.2987	0.7872	0.8494	0.086*	

### Atomic displacement parameters (Å^2^)

**Table:**

	*U*^11^	*U*^22^	*U*^33^	*U*^12^	*U*^13^	*U*^23^
S1	0.0378 (6)	0.0369 (6)	0.0381 (6)	−0.0007 (6)	0.0260 (5)	−0.0026 (5)
O1	0.068 (2)	0.0397 (19)	0.0475 (19)	0.0079 (17)	0.0404 (19)	0.0108 (15)
O2	0.0334 (17)	0.054 (2)	0.0472 (18)	−0.0044 (15)	0.0267 (15)	−0.0123 (15)
O3	0.055 (2)	0.079 (3)	0.064 (2)	−0.0214 (19)	0.046 (2)	−0.0109 (19)
N1	0.045 (2)	0.041 (2)	0.038 (2)	−0.0101 (18)	0.0304 (19)	−0.0067 (17)
C1	0.036 (2)	0.033 (3)	0.034 (2)	0.001 (2)	0.024 (2)	0.0004 (19)
C2	0.046 (3)	0.041 (3)	0.061 (3)	−0.008 (2)	0.040 (3)	−0.005 (2)
C3	0.058 (3)	0.035 (3)	0.067 (3)	−0.009 (2)	0.046 (3)	−0.006 (2)
C4	0.045 (3)	0.042 (3)	0.034 (2)	0.009 (2)	0.026 (2)	0.002 (2)
C5	0.048 (3)	0.052 (3)	0.062 (3)	−0.004 (3)	0.045 (3)	−0.005 (3)
C6	0.053 (3)	0.037 (3)	0.063 (3)	−0.006 (2)	0.046 (3)	−0.008 (2)
C7	0.036 (3)	0.038 (3)	0.045 (3)	−0.007 (2)	0.026 (2)	−0.002 (2)
C8	0.024 (5)	0.017 (5)	0.056 (6)	0.010 (4)	0.030 (5)	0.005 (4)
C9	0.034 (5)	0.032 (6)	0.035 (5)	0.001 (4)	0.026 (5)	0.001 (4)
C10	0.024 (5)	0.038 (6)	0.041 (5)	0.007 (4)	0.026 (5)	0.000 (4)
C11	0.028 (5)	0.036 (5)	0.036 (6)	0.006 (4)	0.018 (5)	0.002 (4)
C12	0.050 (7)	0.035 (5)	0.063 (7)	0.006 (5)	0.046 (6)	−0.006 (4)
C13	0.041 (7)	0.047 (6)	0.043 (6)	−0.006 (6)	0.032 (6)	−0.011 (5)
Cl1	0.0510 (16)	0.0559 (16)	0.0613 (16)	0.0102 (12)	0.0392 (14)	0.0080 (12)
Cl1'	0.078 (2)	0.065 (2)	0.110 (3)	−0.0042 (18)	0.069 (2)	−0.0242 (19)
C14	0.066 (3)	0.054 (3)	0.055 (3)	0.017 (3)	0.041 (3)	0.005 (3)

### Geometric parameters (Å, º)

**Table:**

S1—O1	1.407 (3)	C9—Cl1	1.745 (9)
S1—O2	1.446 (3)	C10—C11	1.397 (12)
S1—N1	1.654 (3)	C10—H10	0.9300
S1—C1	1.754 (4)	C11—C12	1.420 (12)
O3—C7	1.195 (5)	C11—H11	0.9300
N1—C7	1.400 (5)	C12—C13	1.342 (11)
N1—H1N	0.8600	C12—H12	0.9300
C1—C2	1.377 (5)	C13—H13	0.9300
C1—C6	1.380 (5)	C8'—C13'	1.373 (17)
C2—C3	1.378 (6)	C8'—C9'	1.412 (15)
C2—H2	0.9300	C9'—C10'	1.423 (16)
C3—C4	1.384 (6)	C9'—Cl1'	1.762 (13)
C3—H3	0.9300	C10'—C11'	1.368 (15)
C4—C5	1.385 (6)	C10'—H10'	0.9300
C4—C14	1.506 (6)	C11'—C12'	1.398 (14)
C5—C6	1.369 (6)	C11'—H11'	0.9300
C5—H5	0.9300	C12'—C13'	1.360 (16)
C6—H6	0.9300	C12'—H12'	0.9300
C7—C8'	1.408 (12)	C13'—H13'	0.9300
C7—C8	1.589 (11)	C14—H14A	0.9600
C8—C13	1.374 (14)	C14—H14B	0.9600
C8—C9	1.419 (12)	C14—H14C	0.9600
C9—C10	1.410 (12)		
			
O1—S1—O2	118.86 (19)	C8—C9—Cl1	126.8 (7)
O1—S1—N1	107.20 (18)	C11—C10—C9	112.3 (8)
O2—S1—N1	105.20 (17)	C11—C10—H10	123.8
O1—S1—C1	110.41 (19)	C9—C10—H10	123.8
O2—S1—C1	108.09 (19)	C10—C11—C12	124.9 (7)
N1—S1—C1	106.31 (18)	C10—C11—H11	117.5
C7—N1—S1	127.5 (3)	C12—C11—H11	117.5
C7—N1—H1N	116.2	C13—C12—C11	121.1 (9)
S1—N1—H1N	116.2	C13—C12—H12	119.5
C2—C1—C6	121.2 (4)	C11—C12—H12	119.5
C2—C1—S1	119.3 (3)	C12—C13—C8	116.7 (11)
C6—C1—S1	119.5 (3)	C12—C13—H13	121.6
C1—C2—C3	119.0 (4)	C8—C13—H13	121.6
C1—C2—H2	120.5	C13'—C8'—C7	122.9 (11)
C3—C2—H2	120.5	C13'—C8'—C9'	121.9 (12)
C2—C3—C4	120.8 (4)	C7—C8'—C9'	114.5 (10)
C2—C3—H3	119.6	C8'—C9'—C10'	119.3 (12)
C4—C3—H3	119.6	C8'—C9'—Cl1'	125.9 (10)
C3—C4—C5	118.7 (4)	C10'—C9'—Cl1'	114.7 (12)
C3—C4—C14	120.1 (4)	C11'—C10'—C9'	114.7 (13)
C5—C4—C14	121.1 (4)	C11'—C10'—H10'	122.7
C6—C5—C4	121.2 (4)	C9'—C10'—H10'	122.7
C6—C5—H5	119.4	C10'—C11'—C12'	126.5 (15)
C4—C5—H5	119.4	C10'—C11'—H11'	116.8
C5—C6—C1	119.0 (4)	C12'—C11'—H11'	116.8
C5—C6—H6	120.5	C13'—C12'—C11'	117.5 (15)
C1—C6—H6	120.5	C13'—C12'—H12'	121.3
O3—C7—N1	123.2 (4)	C11'—C12'—H12'	121.3
O3—C7—C8'	115.9 (7)	C12'—C13'—C8'	119.7 (13)
N1—C7—C8'	120.3 (7)	C12'—C13'—H13'	120.1
O3—C7—C8	125.2 (5)	C8'—C13'—H13'	120.1
N1—C7—C8	111.3 (5)	C4—C14—H14A	109.5
C8'—C7—C8	15.5 (7)	C4—C14—H14B	109.5
C13—C8—C9	122.8 (9)	H14A—C14—H14B	109.5
C13—C8—C7	120.6 (8)	C4—C14—H14C	109.5
C9—C8—C7	116.5 (8)	H14A—C14—H14C	109.5
C10—C9—C8	122.0 (8)	H14B—C14—H14C	109.5
C10—C9—Cl1	111.1 (6)		

### Hydrogen-bond geometry (Å, º)

**Table:**

*D*—H···*A*	*D*—H	H···*A*	*D*···*A*	*D*—H···*A*
N1—H1*N*···O2^i^	0.86	2.02	2.867 (4)	169

Symmetry code: (i) −*x*+1, *y*, −*z*+3/2.
